# A bibliometric analysis of chemotherapy and pain in pediatric patients over the last decade

**DOI:** 10.3389/fped.2023.1269736

**Published:** 2023-12-08

**Authors:** Hua Huang, Guowei Cai, Hongchun Xiang

**Affiliations:** ^1^ Department of Pediatric Hematology, Union Hospital, Tongji Medical College, Huazhong University of Science and Technology, Wuhan, China; ^2^ Department of Acupuncture and Moxibustion, Union Hospital, Tongji Medical College, Huazhong University of Science and Technology, Wuhan, China

**Keywords:** chemotherapy, cancer, children, pain, CiteSpace

## Abstract

**Background:**

Chemotherapy is an important treatment for children with cancer, and chemotherapy-induced pain is an important role in affecting patients' quality of life. In our study, bibliometric analysis was used to identify current research hotspots and future research trends of chemotherapy and pain in children over the last decade. Our findings can provide a reference for the research in the field of chemotherapy and pain in children.

**Method:**

Publications of chemotherapy and pain in children were collected from the Web of Science Core Collection database. CiteSpace was used to analyze publication characteristics from 2013 to 2022.

**Results:**

We identified 1,130 eligible publications in the field of chemotherapy and pain in children, with an increasing trend of publications over the last decade. In the field of chemotherapy and pain in children, the United States had the most publication with 346, followed by China with 135. The author with the most published papers was Pamela S Hinds (*n* = 8) from the United States. The journals that published the most papers were the Journal of pediatric hematology oncology (*n* = 44) and Medicine (*n* = 44). The Journal of Clinical Oncology was cited the most frequency (*n* = 422). St. Jude Children's Research Hospital had the most publication (*n* = 23). The specific keywords related to the field of chemotherapy and pain in children were “children”, “chemotherapy”, “management”, “childhood cancer”, “randomized controlled trial” and “efficacy”. Emerging research focuses predominantly on symptomatic and supportive interventions for chemotherapy and pain in children.

**Conclusion:**

Attention to chemotherapy and pain in children with cancer was insufficient. This bibliometric analysis showed the upward trend of chemotherapy and pain in children over the last decade. More studies are needed to improve the quality of life in children with chemotherapy-induced pain. This study may provide useful information to guide future research on chemotherapy and pain in children.

## 1. Introduction

Pediatric chemotherapy-induced pain comprises a series of painful symptoms caused by chemotherapeutic agents (1). For instance, vincristine (VCR) can destroy microtubules in peripheral nerves and enhance inflammatory processes and axonal dysfunction, which may cause neuropathic pain, paresthesia, and numbness (2). Meanwhile, the infiltration of immune inflammatory cells is an important cause of pain in VCR-induced peripheral neuropathy (3). In infant, chemotherapy-induced peripheral neuropathy significantly decreases the quality of life (QoL) of cancer patients, because the peripheral nervous system in infancy is still developing, and nerve damage can lead to significant hyperalgesia in adulthood (4). A previous study has shown that VCR can cause peripheral neuropathy, and the injured nerve fibers abnormally transmit signals to the pain center, which induces different degrees of neuropathic pain (5). Among patients with acute lymphoblastic leukemia, the majority of pediatric patients (84%) receiving chemotherapy experiences one painful event, and neuropathic pain may be a risk factor for learning problems after completion of treatment (6). Methotrexate can induce stomatitis, which induces mouth pain and discomfort (7). The research on adulthood chemotherapy-induced pain showed the characteristics of multi-fields, multi-perspectives, and stable development (8), but further studies on pediatric chemotherapy-induced pain are needed (9).

With the continuous progress of medical technology, the survival rate of more and more cancer survivors has been improved, and pediatric chemotherapy-induced pain has become a rising health concern (10). However, clinical trials studying the prevention and treatment of pain and related symptoms in pediatrics are rare, which necessitates research on pediatric chemotherapy and pain. Therefore, this study mainly analyzed publications related to chemotherapy and pain in children, and explored the research trends and hot spots of this field.

Bibliometric analysis analyzes citation data and assesses the academic impact of relevant literature in a given field. Meanwhile, key research and topics that influence disease management and knowledge are identified through quantitative analysis (11). In addition, bibliometric analysis can accurately identify the contributions of authors, journals, institutions, and countries to specific research direction, and predict the research trend in a certain field (12). There is a significant body of literature on pediatric chemotherapy and pain, such as bleomycin for pediatric lymphangiomas (13), mercaptopurine for acute lymphoblastic leukemia (14) and irinotecan plus doxorubicin for refractory pediatric Wilms tumor (15). However, the extensive literature in this field has not been systematically classified and sorted out. Therefore, we conducted a bibliometric analysis of publications related to chemotherapy and pain in children from 2013 to 2022. We aimed to elucidate existing research hotspots and potential trends, thereby providing useful reference guidance for future research.

## 2. Data collection and research methods

### 2.1. Data source and collection

We screened the Web of Science Core Collection (WoSCC) database from January 1, 2013 to December 31, 2022 to identify publications about pediatric chemotherapy and pain. The search formula was set to TS = ((chemotherapy) OR (antineoplastic agent*) OR (oxaliplatin) OR (paclitaxel) OR (docetaxel) OR (vinorelbine) OR (bortezomib) OR (vinc*) OR (cisplatin) OR (taxane) OR (doxorubicin) OR (Pirarubicin) OR (Cyclophosphamide) OR (Dactinomycin) OR (Bleomycin A5 Hydrochloride) OR (fluorouracil) OR (Cytarabine) OR (mercaptopurine) OR (Gemcitabine) OR (fludarabine) OR (Methotrexate) OR (camptothecin) OR (anthracycline) OR (etoposide) OR (Alkylating agent) OR (Isocyclophosphamide) OR (carboplatin) OR (bleomycin) OR (Bortezomib) OR (Arsenic trioxide) OR (Retinoic acid*) OR (L-asparaginase) OR (Rituximab) OR (Activin Like Kinase inhibitor*) OR (Tyrosine kinase inhibitor*) OR (Neurotrophic factor receptor tyrosine kinase inhibitor*)) AND TS = ((pediatric*)OR (child*) OR (kid) OR (kids) OR (baby) OR (babies) OR (infant*) OR (toddler*)) AND TS = (pain), period = (2013–2022).

### 2.2. Bibliometric analysis

CiteSpace v.6.1.R6 was used for bibliometric analysis. In this study, each node represented an aspect, such as country, institution, author, journal, publications and keyword.

The size of node indicated the number of publications or the frequency with which they are cited. The greater the centrality value of network nodes, the more important the influence of nodes in the network (16). In different maps, the timeline is displayed on the left side of the network visualization map, with different link colors representing different times. According to concurrency analysis, the keywords can be divided into different clusters with different colors.

## 3. Results

### 3.1. Analysis of basic information

In this study, 1,130 publications that met the criteria were analyzed from 2013 to 2022. The number of publications in pediatric chemotherapy and pain maintained a gradually increasing trend and attention from 2013 to 2022.

#### 3.1.1. Country/region analysis

**Figure 1:**
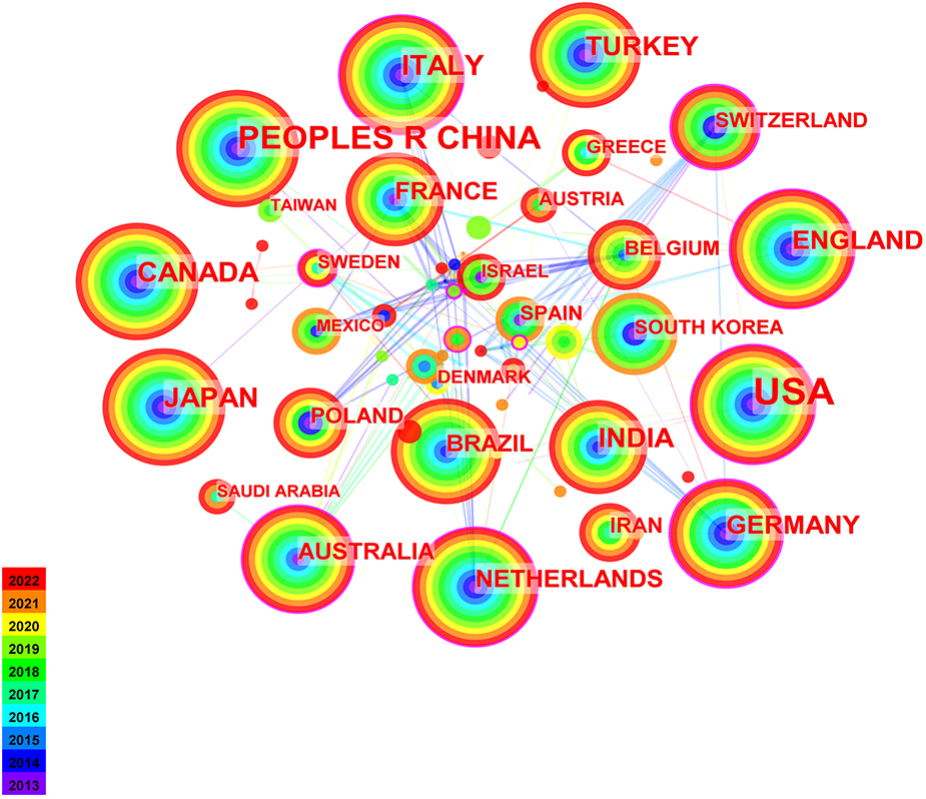
Network of the productive countries on pediatric chemotherapy-related pain.

In Figure 1, the content is national collaborative network for pediatric chemotherapy and pain studies. In Table 1, the United States produced the most publications (*n* = 346), followed by China (*n* = 135), Italy (*n* = 70), Japan (*n* = 67), and Canada (*n* = 64). Centrality indicates the importance of nodes in a network. According to centrality, Germany (0.35) was the core of the network, followed by Switzerland (0.27) and South Africa (0.27). In collaborative networks, higher centrality implies more frequent cooperation.

**Table 1:** Rank of the number and centrality of papers published by countries.

Rank	Publication	Centrality	Countries/regions	Rank	Centrality	Publication	Countries/regions
1	346	0.19	USA	1	0.35	46	GERMANY
2	135	0.03	PEOPLES R CHINA	2	0.27	20	SWITZERLAND
3	70	0.12	ITALY	3	0.27	6	SOUTH AFRICA
4	67	0	JAPAN	4	0.27	5	CZECH REPUBLIC
5	64	0.07	CANADA	5	0.22	5	RUSSIA
6	59	0	INDIA	6	0.2	33	AUSTRALIA
6	57	0	TURKEY	7	0.19	346	USA
8	49	0.11	ENGLAND	8	0.14	14	SWEDEN
8	46	0.35	GERMANY	9	0.13	37	NETHERLANDS
10	37	0.03	FRANCE	10	0.12	70	ITALY
10	37	0.13	NETHERLANDS	10	0.12	8	NORWAY

#### 3.1.2. Institutional analysis

**Figure 2:**
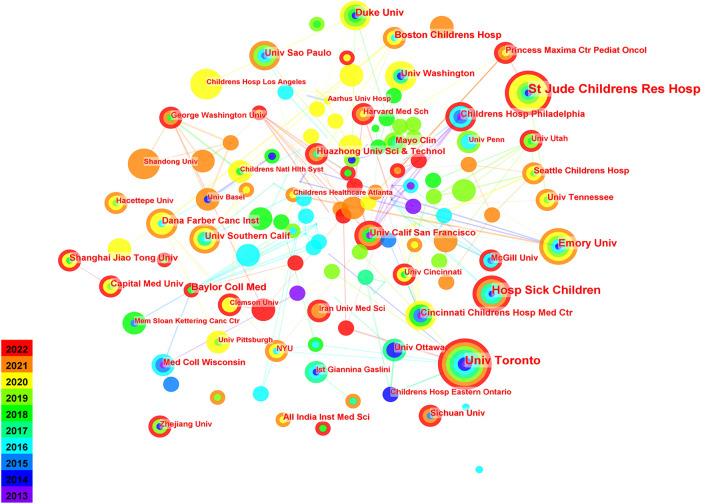
Network of the productive institutions.

The profile of institutions consisted of 322 nodes and 507 linear connections (Figure 2). Over the past decade, 322 institutions have involved in the pediatric chemotherapy and pain, with St. Jude Children's Research Hospital (*n* = 23) producing the most publications, followed by University of Toronto (*n* = 20), and Hospital for Sick Children (*n* = 18) (Table 2). In terms of centrality (Table 2), at the top of the centrality ranking was Clemson University (0.1), followed by University of California, San Francisco (0.09) and New York University (centrality = 0.09). As shown in Figure 2, there was extensive inter-agency collaboration. The most productive institutions were St. Jude Children's Research Hospital, University of Toronto, Hospital for Sick Children, and Emory University.

**Table 2:** Rank of the number and centrality of papers published by institutions.

Rank	Publications	Centrality	Institutions	Rank	Publications	Centrality	Institutions
1	23	0.05	St Jude Childrens Res Hosp	1	5	0.1	Clemson Univ
2	20	0.05	Univ Toronto	2	8	0.09	Univ Calif San Francisco
3	18	0	Hosp Sick Children	3	6	0.09	NYU
4	13	0.05	Emory Univ	4	23	0.05	St Jude Childrens Res Hosp
5	11	0.05	Duke Univ	5	20	0.05	Univ Toronto
6	11	0.03	Baylor Coll Med	6	13	0.05	Emory Univ
7	10	0.02	Boston Childrens Hosp	7	11	0.05	Duke Univ
8	9	0	Univ Southern Calif	8	9	0.05	Dana Farber Canc Inst
9	9	0.03	Cincinnati Childrens Hosp Med Ctr	9	7	0.05	Princess Maxima Ctr Pediat Oncol
10	9	0.01	Childrens Hosp Philadelphia	10	7	0.04	Seattle Childrens Hosp

#### 3.1.3. Authors and co-cited authors analysis

**Figure 3:**
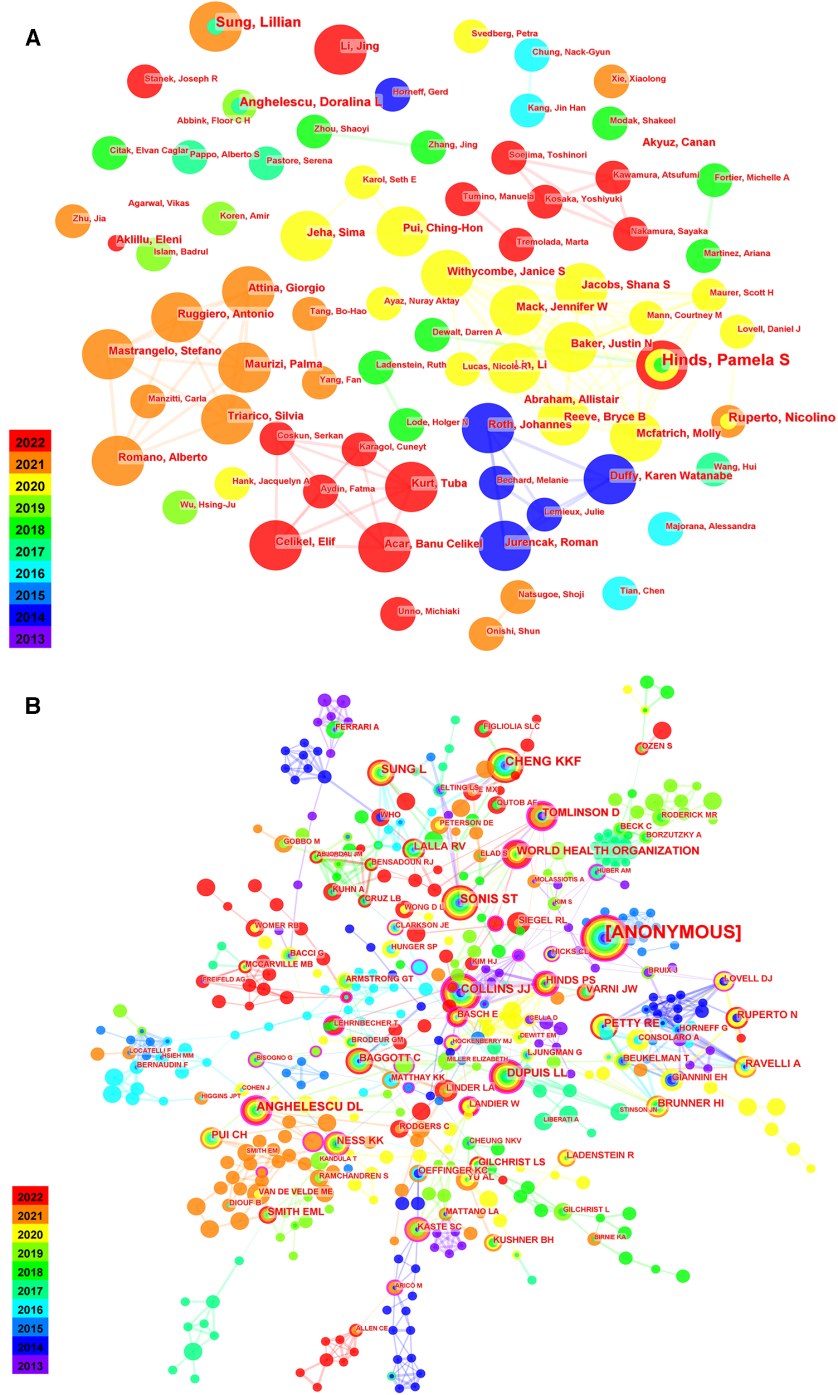
Co-cited authors of research on chemotherapy and pain in children. (**A**) Map of authors network. (**B**) Map of co-cited authors network.

Over the past decade, two hundred authors contributed to the publications related to pediatric chemotherapy and pain. Among them, the most published authors were Pamela S Hinds (*n* = 8) (17). In the top 10 citation co-cited authors, CHENG KKF (*n* = 34) (18) ranked first, followed by SONIS ST (n = 33) and ANGHELESCU DL (*n* = 30). From the centrality view (Table 3), COLLINS JJ (centrality = 0.38) ranked first, followed by ANGHELESCU DL (centrality = 0.27) and DUPUIS LL (centrality = 0.27). In the network between authors or co-cited authors (Figure 3), each node means an author. Regarding authors' links or co-cited authors' view, the size of a node means the number of publications or the number of times being cited. More publications or citations were shown by larger nodes. A few authors collaborated with each other, suggesting that better collaboration may advance the field.

**Table 3:** Top 10 citation and centrality of co-cited authors on chemotherapy and pain in children.

Rank	Citation	Centrality	Co-cited author (affiliation)	Rank	Centrality	Citation	Co-cited Author (affiliation)
1	34	0.03	CHENG KKF	1	0.38	27	COLLINS JJ
2	33	0.08	SONIS ST	2	0.27	30	ANGHELESCU DL
3	30	0.27	ANGHELESCU DL	3	0.27	22	DUPUIS LL
4	27	0.38	COLLINS JJ	4	0.24	2	BLOOM BJ
5	25	0.01	SUNG L	5	0.23	5	HOCKENBERRY MJ
6	24	0.16	WORLD HEALTH ORGANIZATION	6	0.22	20	TOMLINSON D
7	22	0.02	PETTY RE	7	0.21	18	HINDS PS
8	22	0.27	DUPUIS LL	8	0.21	5	HUBER AM
9	20	0.22	TOMLINSON D	9	0.2	5	BISOGNO G
10	18	0.08	BRUNNER HI	10	0.2	4	JACOB EUFEMIA

#### 3.1.4. Journals and cited journals analysis

**Figure 4:**
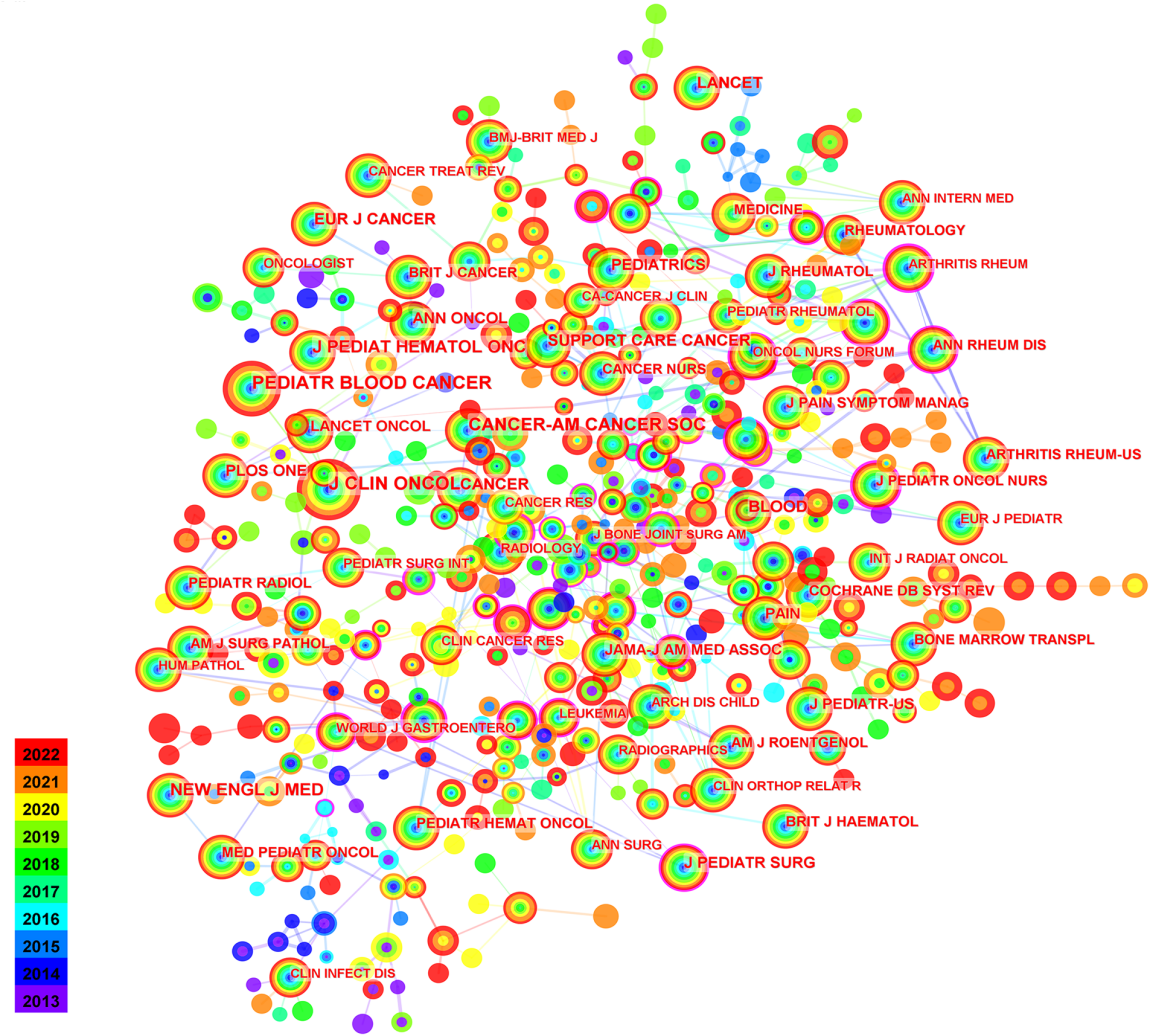
Analysis of cited journals. Work and network map of the most active cited journals in the field of pediatric chemotherapy-related pain from 2013 to 2022.

In the last decade, the three journals that published the most papers in the field of pediatric chemotherapy and pain were *Journal of pediatric hematology oncology* (*n* = 44), *Medicine* (*n* = 44), and *Pediatric blood & cancer* (*n* = 41). According to the cited journals (Figure 4), the 10 most journals with cited frequency and highest centrality of the field of chemotherapy and pain in children are listed in Table 4. The three most cited journals were the *Journal of Clinical Oncology* with 422 citations, *Pediatric Blood & Cancer* (*n* = 388) and *Cancer-American Cancer Society* (*n* = 304). In terms of centrality, *Archives of Rheumatology* ranked first with a centrality of 0.31, and in citation frequency with 58 citations. *The Journal of clinical investigation* ranked second with a centrality of 0.28 and in citation frequency with 28 citations.

**Table 4:** Top 10 most cited journals on chemotherapy and pain in children.

Rank	Frequnecy	Centrality	Cited journal	Rank	Centrality	Frequnecy	Cited journal
1	422	0.08	J CLIN ONCOL	1	0.31	58	ARTHRITIS RHEUM
2	388	0.01	PEDIATR BLOOD CANCER	2	0.28	33	J CLIN INVEST
3	304	0.02	CANCER-AM CANCER SOC	3	0.26	31	AM J PATHOL
4	284	0	NEW ENGL J MED	4	0.26	27	EUR J PEDIATR SURG
5	259	0	J PEDIAT HEMATOL ONC	5	0.25	90	J PEDIATR ONCOL NURS
6	190	0.03	CANCER	6	0.22	8	J NEUROSCI
7	186	0	LANCET	7	0.21	50	ARCH PATHOL LAB MED
8	180	0	BLOOD	8	0.2	48	J NEURO-ONCOL
9	171	0	EUR J CANCER	9	0.19	44	LEUKEMIA LYMPHOMA
10	166	0.01	SUPPORT CARE CANCER	10	0.18	32	J PEDIATR ORTHOPED

### 3.2. Progress on pediatric chemotherapy and pain based on literature analysis

#### 3.2.1. Analysis of publications co-citation and burst

Literature co-citation is when 2 or more publications are cited simultaneously by one or more articles. Through the co-citation relationship between the two publications in the map, we can dig out the content of common interest and clarify the close relationship between two publications (19). Through citation burst analysis, publications with high citation growth rate can be selected and tracked. A high citation growth rate means that an article has been cited rapidly in the last few years, which indicates new changes in the field of research (20). Thus, we analyzed the core content in pediatric chemotherapy and pain over the last decade.

**Figure 5:**
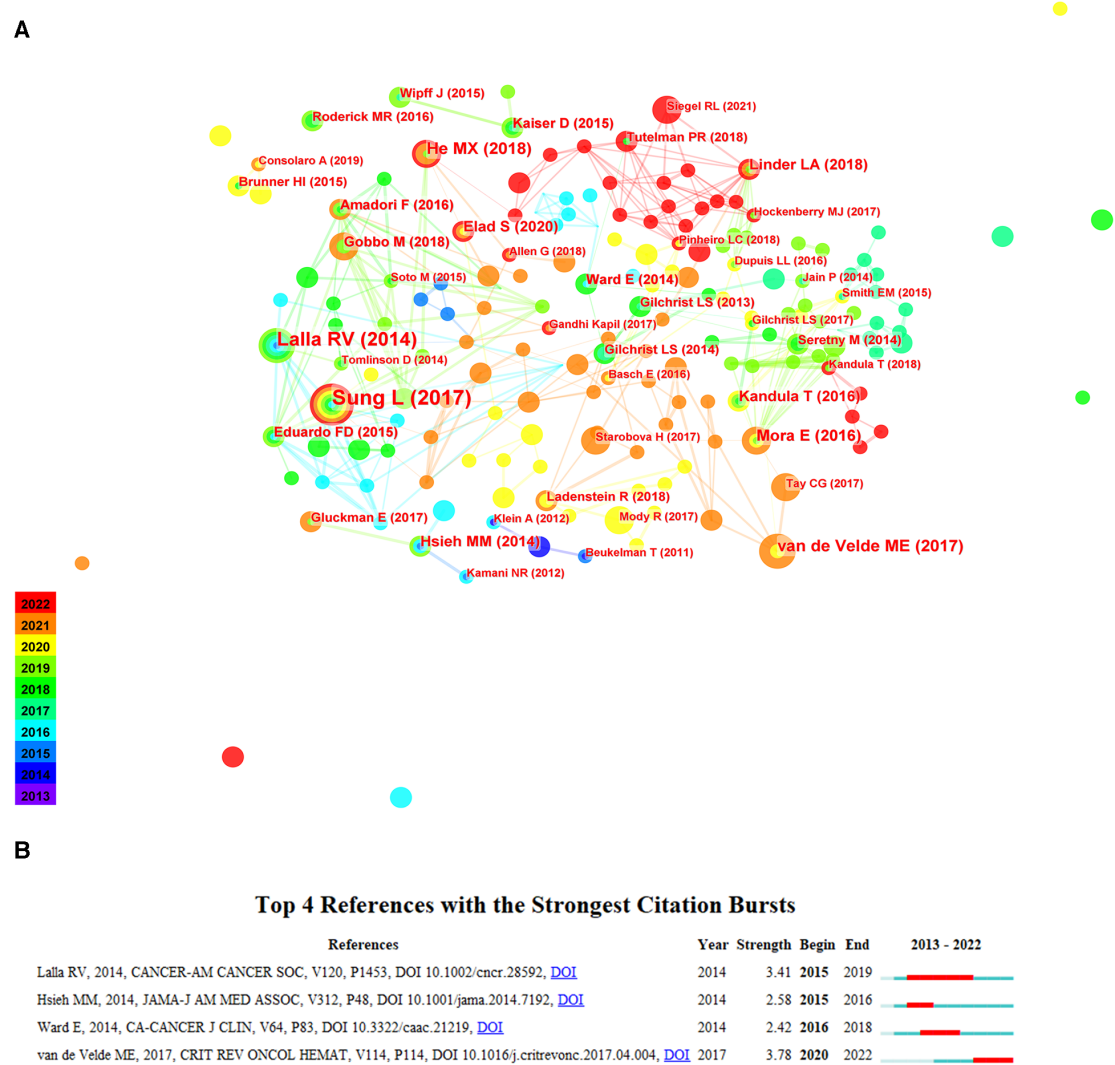
The links map and cluster map of co-cited references. (**A**) The links of co-cited references of chemotherapy and pain in children. (**B**) Top 4 references with the strongest citation bursts of chemotherapy and pain in children.

Reference maps for chemotherapy and pain in children were identified in the literature from 2013 to 2022. Nodes on the map represent different references. The purple area appears earlier than the green area, and the green area appears later than the red area. Again, the size of each node means the frequency at which it is referenced, and the innermost color is the time when it was first referenced (Figure 5A). Figure 5B shows the top 4 most frequently cited articles. According to Figure 5B, the strongest citation bursts lasted until 2022 with one article. The article is entitled “Vincristine-induced peripheral neuropathy in children with cancer: A systematic review”. It concluded that VCR has been used as a chemotherapeutic agent for treating many types of cancers in adult and children. VCR-induced peripheral neuropathy (VIPN) can lead to decreased motor function or pain in children, which affects the QoL (21). There was one paper in the top 4 references with the strongest citation bursts that lasted more than 5 years. The article is entitled “MASCC/ISOO clinical practice guidelines for the management of mucositis secondary to cancer therapy”. Severe secondary catarrh caused by chemotherapy may require reduction of chemotherapy dose or interruption of treatment. The Multinational Association of Supportive Care in Cancer and International Society of Oral Oncology (MASCC/ISOO) will assist clinicians in the evidence-based management of mucositis secondary to cancer treatment (22).

In Table 5, the 10 most frequently cited papers are shown. In terms of centrality, the top 10 cited documents are listed in Table 6. The article entitled “Guideline for the prevention of oral and oropharyngeal mucositis in children receiving treatment for cancer or undergoing haematopoietic stem cell transplantation” concluded that oral and oropharyngeal mucositis is a common side effect of chemotherapy in adults and children with cancer. Oral mucositis can lead to severe mouth and throat ulcers, causing pain to eat and drink, and sometimes requiring hospitalization for rehydration or parenteral nutrition (7). This paper has been cited most frequently and ranks first based on centrality. It indicates the importance of peer researchers' attention to chemotherapy-induced oral mucositis in the past 10 years and determines the direction of future research.

**Table 5:** The 10 most frequently cited papers of chemotherapy and pain in children.

Rank	Frequency	Reference
1	20	Sung L, 2017, BMJ SUPPORT PALLIAT, V7, P7, DOI 10.1136/bmjspcare-2014-000804
2	15	Lalla RV, 2014, CANCER-AM CANCER SOC, V120, P1453, DOI 10.1002/cncr.28592
3	9	He MX, 2018, EUR J PEDIATR, V177, P7, DOI 10.1007/s00431-017-3043-4
4	9	van de Velde ME, 2017, CRIT REV ONCOL HEMAT, V114, P114, DOI 10.1016/j.critrevonc.2017.04.004
5	8	Mora E, 2016, AM J CANCER RES, V6, P2416
6	7	Linder LA, 2018, CANCER NURS, V41, P23, DOI 10.1097/NCC.0000000000000469
7	7	Kandula T, 2016, CANCER TREAT REV, V50, P118, DOI 10.1016/j.ctrv.2016.09.005
8	7	Elad S, 2020, CANCER-AM CANCER SOC, V126, P4423, DOI 10.1002/cncr.33100
9	7	Hsieh MM, 2014, JAMA-J AM MED ASSOC, V312, P48, DOI 10.1001/jama.2014.7192
10	6	Gobbo M, 2018, PEDIATR BLOOD CANCER, V65, P0, DOI 10.1002/pbc.27098

**Table 6:** Rank of the cited references with the highest centrality of chemotherapy and pain in children.

Rank	Centrality	Reference
1	0.07	Mora E, 2016, AM J CANCER RES, V6, P2416
2	0.06	Sung L, 2017, BMJ SUPPORT PALLIAT, V7, P7, DOI 10.1136/bmjspcare-2014-000804
3	0.06	He MX, 2018, EUR J PEDIATR, V177, P7, DOI 10.1007/s00431-017-3043-4
4	0.06	Loprinzi CL, 2020, J CLIN ONCOL, V38, P0, DOI 10.1200/JCO.20.01399
5	0.05	Ward E, 2014, CA-CANCER J CLIN, V64, P83, DOI 10.3322/caac.21219
6	0.05	Starobova H, 2017, FRONT MOL NEUROSCI, V10, P0, DOI 10.3389/fnmol.2017.00174
7	0.05	Geisler S, 2016, BRAIN, V139, P3092, DOI 10.1093/brain/aww251
8	0.04	Tutelman PR, 2018, CLIN J PAIN, V34, P198, DOI 10.1097/AJP.0000000000000531
9	0.04	Anschau F, 2019, LASER MED SCI, V34, P1053, DOI 10.1007/s10103-019-02722-7
10	0.03	Linder LA, 2018, CANCER NURS, V41, P23, DOI 10.1097/NCC.0000000000000469

### 3.3. Keywords analysis of progress on chemotherapy and pain of children

#### 3.3.1. Keywords' frequency and cluster

**Figure 6:**
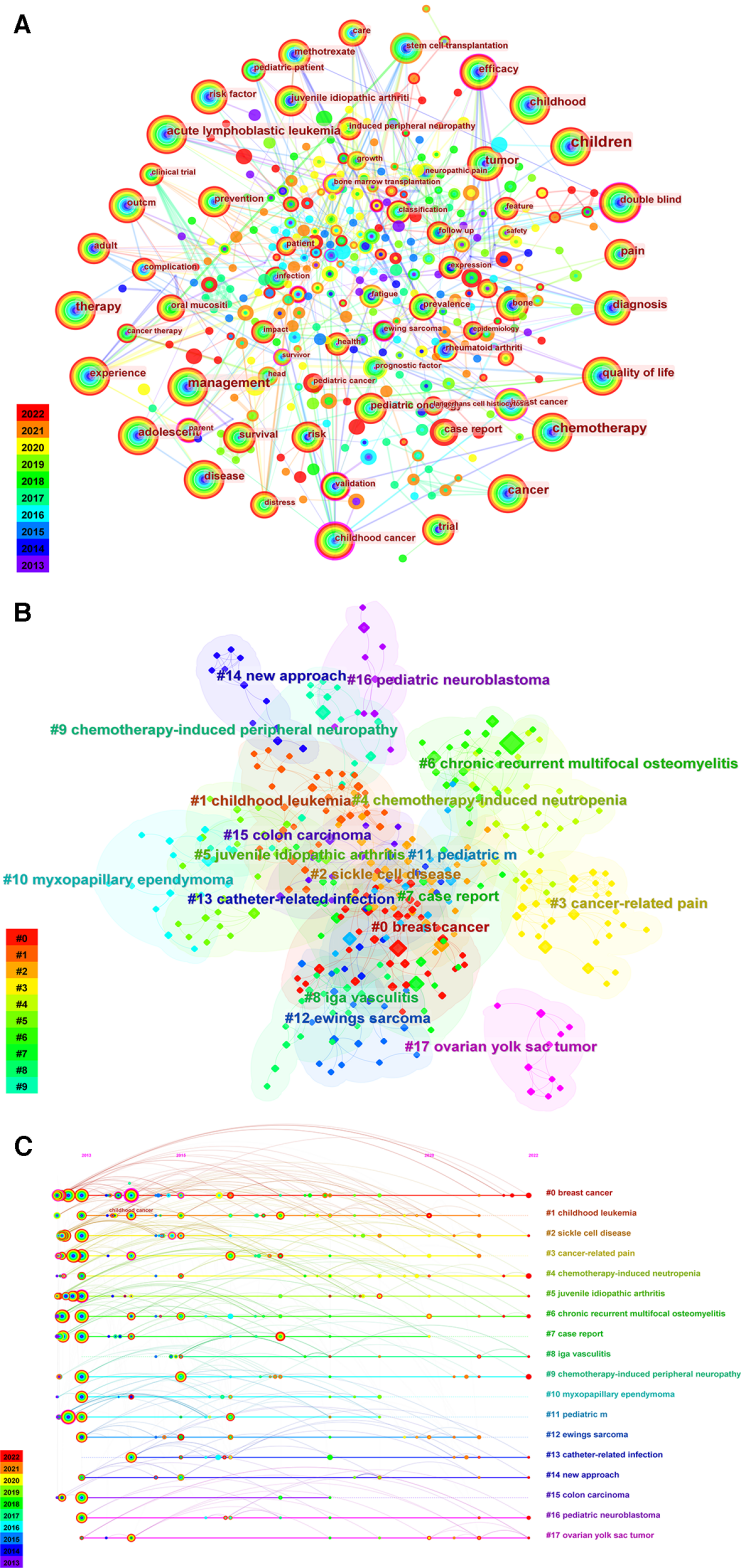
Analysis of keywords. (**A**) Co-occurrence graph of the keywords. (**B**) Analysis of keywords cluster map in chemotherapy-related pain studies. (**C**) The chronological order in which the keyword appears in each cluster.

The research hotspot refers to scientific problems that are concerned by numerous scholars in a certain field at a given time. Keywords are the refined expression of the research topic and represent the content of academic papers. The keywords co-occurrence network reflects the current research hotspots of a certain field over the past decade (23). Figure 6A shows the keywords co-occurrence graph. This graph shows the hotspots in the literature related to chemotherapy and pain in children with cancer.

We analyzed the top co-occurrence frequency and centrality keywords related to chemotherapy and pain in children over the past decade (Table 7). Co-occurrence frequency refers to the frequency of keywords occurrence. Centrality means the degree to which a keyword is paid attention. “Children” was the most frequently used word at 333, followed by “chemotherapy” at 138 and “management” at 130. In terms of centrality, the top 3 keywords were “childhood cancer” (0.21), “randomized controlled trial” (0.18) and “efficacy” (0.16).

**Table 7:** Top 10 keywords related to chemotherapy and pain of children.

Rank	Frequnecy	Centrality	Keyword	Rank	Centrality	Frequnecy	Keyword
1	333	0.01	Children	1	0.21	29	Childhood cancer
2	138	0.01	Chemotherapy	2	0.18	6	Randomized controlled trial
3	130	0.03	Management	3	0.16	42	Efficacy
4	93	0	Therapy	4	0.15	22	Validation
5	91	0.03	Cancer	5	0.15	20	Breast cancer
6	79	0.03	Acute lymphoblastic leukemia	6	0.15	15	Ewing sarcoma
7	73	0.08	Quality of life	7	0.15	12	Survivor
8	71	0.01	Pain	8	0.14	8	Anxiety
9	70	0.04	Adolescent	9	0.12	17	Parent
10	69	0.07	Diagnosis	10	0.11	6	Lymphoma

The highlighted keywords in Figure 6A are represented by nodes, indicating that the co-occurrence frequency greatly changed between 2013 and 2022. Graphs drawn by CiteSpace software can be measured by *Q* value and *S* value. Modularity is measured by *Q* value. *Q* values greater than 0.3 indicate the significance of the cluster structure. The value of *S* is Silhouette. Clustering with *S* > 0.5 is generally considered reasonable, and clustering with *S* > 0.7 is valid and persuasive (19). As shown in Figure 6B, 18 main keywords were obtained by visualization diagram analysis, whose *Q* value was 0.7297, indicating that the cluster structure is significant and the clustering effect is good. *S* value was 0.8823, suggesting that the clustering results are certain. High-quality clustering analysis are helpful to explore the overall characteristics and development trend of chemotherapy and pain in children according to keywords in the timeline of the cluster map. The chronological order in which the keywords appeared in each cluster is displayed in Figure 6C.

#### 3.3.2. Analysis of keywords' burst

**Figure 7:**
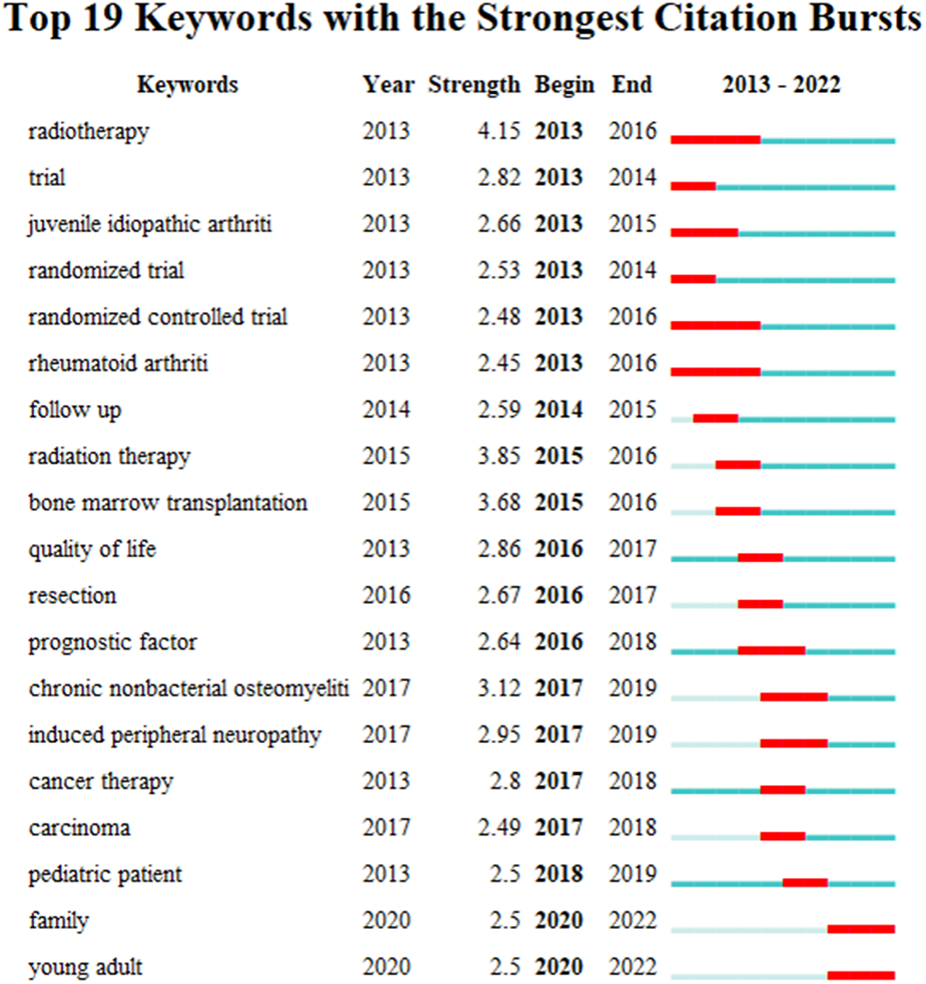
The top 19 most frequently cited keywords with the strongest citation bursts.

We used CiteSpace to generate the top 19 strongest burst keywords (Figure 7), such as “radiotherapy”, “bone marrow transplantation” and “radiation therapy”. Both chemotherapy and radiation can induce peripheral neuropathy. It is becoming increasingly important to improve the QoL after treating childhood cancer patients. Additional studies are needed to optimize treatment options and reduce pain and dysfunction related to treatment toxicity in pediatric cancer (24).

## 4. Discussion

Chemotherapeutics may cause sensory, motor, and/or autonomic neuropathy, which may result in lower chemotherapeutic dose, delay or discontinuation of chemotherapy. Meanwhile, chemotherapy-induced neuropathy can also cause pain and dysfunction, which can reduce QoL and increase medical costs (25). The main toxicity of VCR is VIPN, which can significantly reduce health-related QoL (HR-QoL), including Pain and Hurt domain of Pediatric Cancer Quality of Life Inventory™ (PedsQL). CIPN has a significant influence on the health of pediatric patients (26). This study is the first study that used the bibliometric method and visual information technology to reveal major research hotspots in chemotherapy and pain among pediatric patients with cancer. In this study, we searched the WoSCC database for publications on chemotherapy and pain among pediatric patients between 2013 and 2022. The overall trend of publications is increasing year by year. It indicates that more and more scholars are paying attention to the pain caused by chemotherapy in pediatric patients.

Judging by the number of publications in the country, the United States had absolute superiority, and the influence of the articles was also very prominent. China ranked second in the number of published papers, but published less than half as many as the United States. There was little cooperation between the USA and China. The top 10 countries published 85.57% of the total publications, suggesting that these countries were mainly involved in this field of chemotherapy and pain among pediatric cancer patients. There was a lack of academic exchange between countries with more publications and countries with fewer publications. St Jude Children's Research Hospital had the most volume of publications, and the second and third institutions are University of Toronto and Hospital for Sick Children. In terms of institutional influence, Clemson University ranked first, followed by New York University and University of California San Francisco. In terms of publication volume and influence, many universities are mostly from the United States in the top 10 institutions. Different countries and institutions should also exchange and share findings on pediatric chemotherapy and pain to advance the field. Countries and institutions should increase academic exchange and cooperation.

The most prolific author was Pamela S Hinds from the USA (27), and the most co-cited author was Karis Kin-Fong Cheng from Singapore (28). Both of them had significant influence in this field of pediatric chemotherapy and pain. Professor Pamela S Hinds is a fellow of the American Academy of Nursing Sciences, and her research focuses on the psychiatric care of adolescents and children patients. From 2018 to 2022, professor Pamela S Hinds published 7 papers in the field of pediatric chemotherapy and pain. These studies focused on physical functioning, pain disturbances, fatigue and psychological stress and so on in pediatric patients undergoing chemotherapy and radiation therapy (17, 29). The research interest of Professor Cheng included the study of non-drug management for multiple symptoms in pediatric cancer patients. In children and adolescents, a study assessed the efficacy of a home-based symptom-management program for several symptoms of chemotherapy-induced, including nausea, vomiting, fatigue and pain, et al. (30).

From 2013 to 2022, the most cited journals on pediatric chemotherapy and pain were the *Journal of clinical oncology*, *Pediatric blood & cancer*, and *Cancer*, and eight of the top 10 cited journals were from the US. In terms of the influence of the cited journals, *Archives of Rheumatolog* ranked first, followed by *Journal of Clinical Investigation* and *American journal of Clinical Pathology*. These journals focus mainly on oncology and pediatrics.

In a certain period of time, the increase in the number of citations and keywords is a sign of hot topics or cutting-edge trends (31). Over the past decade, we found that “children”, “chemotherapy”, and “management” were the most popular keywords, which can be explained by epidemiology and chemotherapy and pain. Meanwhile, “radiotherapy”, “radiation therapy”, and “bone marrow transplantation” were the top three keywords with the strongest citation bursts. In some cases, inadequate pain treatment during chemotherapy of children can lead to sensitivity to painful stimuli and pain persistence after treatment, thus affecting QoL. The study highlights the importance of education and family member training (32). Children with cancer who receive chemotherapy suffer from a group of psychoneurotic symptoms (PNS) that include symptoms such as pain, anxiety, fatigue, and depression. Metabolomics shows that PNS is associated with fatty acid and amino acid pathways, which could help to develop precise intervention measure to mitigate the effects of PNS on children's QoL (33). An effective analgesic may reduce the pain caused by chemotherapy-induced mucositis. In a retrospective study, adequate analgesia was achieved with tramadol alone (63%) or tramadol combined with morphine (28%). However, 11 percent of patients who received tramadol experienced side effects, compared with 40 percent of those who received morphine as an additive drug (34).

Clinical studies on neuropathy induced by different chemotherapeutic agents have shown that glutamine, duloxetine and venlafaxine are effective in adults. Further more, personalized home exercise programs, therapeutic exercise and aerobic training can improve pain and QoL in chemotherapy patients (35). However, for pediatric chemotherapy patients, there are few studies on the effective treatment of chemotherapy-induced pain, and the relevant studies mainly evaluate pain as a symptom. In particular, basic research on chemotherapy-induced pain is also scarce in pediatric cancer patients, leading to a lack of understanding of its mechanisms. In the future, we need to further explore approaches, including drugs, rehabilitation training, physiotherapy, and psychological intervention, to treat chemotherapy-induced pain in infant. In children with chemotherapy-induced pain, in-depth study of its neurobiological mechanism and multi-center randomized controlled clinical trials for effective treatment of pain are very necessary.

Our study has some limitations. First, this study summarized the hot research topics related to chemotherapy-induced pain in childhood with cancer, but did not analyze each topic in detail. The bibliometric analysis could not reveal details about the type of pain reported (headache, neuropathy, abdominal pain, bone pain, oral pain, etc.), and its range of incidence, including the type of cancer, the age of the patient, the ratio of men to women and cancer-related pain, and whether pediatric cancer patients also received additional radiation therapy. Second, we only analyzed the English-language publications in the WoSCC database and excluded non-English articles. We may have overlooked some important research in other languages, leading to incomplete results. However, these inherent limitations are specific to bibliometric analysis reviews. Other types of review studies are needed to compensate for these limitations.

## 5. Conclusion

This study analyzed research progress, research hotspots, and future directions for pediatric chemotherapy and pain over the past decade. The majority of publications came from the United States. We are confident that more and more countries will be involved in this area of research, and exchanges among countries and institutions will occur more frequently. With the increase in the survival rate of pedriatic patients with cancer, more attention should be paid to the importance of chemotherapy-induced pain on the QoL of children. Future studies will continue to focus on the importance of chemotherapy and pain on the QoL in pediatric cancer patients.
